# Analysis of PD-1, PD-L1, and T-cell infiltration in angiosarcoma pathogenetic subgroups

**DOI:** 10.1007/s12026-021-09259-4

**Published:** 2022-01-19

**Authors:** T. Tomassen, M. E. Weidema, M. H. S. Hillebrandt-Roeffen, C. van der Horst, PALGA group*, I. M. E. Desar, U. E. Flucke, Yvonne M. H. Versleijen-Jonkers

**Affiliations:** 1 Department of Pathology, Radboud University Medical Center, Nijmegen, The Netherlands; 2 Department of Medical Oncology (Internal Postal Code: 452), Radboud University Medical Center, P.O. Box 9101, 6500 HB Nijmegen, The Netherlands; 3 Princess Máxima Center for Pediatric Oncology, Utrecht, The Netherlands

**Keywords:** Angiosarcoma, Subgroups, Programmed cell death 1, Programmed death-ligand 1 (PD-L1), Immune checkpoint inhibition

## Abstract

Angiosarcoma (AS) is a rare malignancy with a poor prognosis. It can develop spontaneously or due to previous radiotherapy (RT), ultraviolet (UV) radiation, or lymphoedema (Stewart Treves AS). Novel therapeutic approaches are needed, but progress is hindered because of the heterogeneity and rarity of AS. In order to explore the potential of immune checkpoint inhibition (ICI), we investigated the protein expression of programmed cell death 1 (PD-1), programmed death-ligand 1 (PD-L1), and CD8 + T cells in 165 AS cases in relation to AS subgroups based on clinical classification and in relation to whole-genome methylation profiling based clusters (A1, A2, B1, B2). High PD-L1 and PD-1 expression were predominantly shown in UV-associated, visceral, and soft tissue AS. RT-associated AS showed predominantly high PD-1 expression. CD8 + T cell infiltration was present in the majority of AS samples. Within the UV-associated AS, two different clusters can be distinguished by DNA methylation profiling. Cases in cluster A1 showed higher PD-1 (*p* = 0.015), PD-L1 (*p* = 0.015), and CD8 + T cells (*p* = 0.008) compared to those in cluster B2, suggesting that these UV-AS tumors are more immunogenic than B2 tumors showing a difference even within one subgroup. In soft tissue AS, combined PD-1 and PD-L1 expression showed a trend toward poor survival (*p* = 0.051), whereas in UV-associated AS, PD-1 expression correlated with better survival (*p* = 0.035). In conclusion, we show the presence of PD-1, PD-L1, and CD8 + T cells in the majority of AS but reveal differences between and within AS subgroups, providing prognostic information and indicating to be predictive for ICI.

**Supplementary Information:**

The online version contains supplementary material available at 10.1007/s12026-021-09259-4.

## Introduction

AS is a rare and aggressive vasoformative sarcoma arising at different anatomical sites, including skin, soft tissue, bone, and visceral organs. AS can be clinically classified into primary AS (with unknown etiology) or secondary AS, in which DNA damaging factors including radiation, UV light exposure or chronic lymphoedema play an important role [1–3]. Current treatment options include surgery, RT, and/or chemotherapy, depending on the extent of the disease. In addition, the multi-tyrosine kinase inhibitor pazopanib is also applied in daily practice [4] and treatment with the generic *ß*-blocker propranolol has been suggested [5]. The survival of AS patients is poor with a reported 5-year survival of only 30–40% [6–8], emphasizing the need for novel treatment options.

A potential approach for AS treatment is ICI. Tumor cells can upregulate PD-L1 on their membrane to promote immune suppression. Interaction with the receptor programmed cell death 1 (PD-1) on CD8 + T cells renders the T cell inactive, and thus prevents the killing of tumor cells. ICI with anti-PD-1 antibodies can reactivate the cytotoxic function of T cells leading to the subsequent killing of tumor cells [9].

In order to determine the role of ICI in AS, it is necessary to examine the expression of PD-L1, PD-1, and the presence of CD8 + T cells as potential biomarkers in this respect. Several studies investigated the expression of these biomarkers in AS, reporting variable levels of expression and varying correlations with prognosis (shown in Table 1). Of note, most studies were performed on cutaneous (predominantly UV-associated) AS or small numbers of other subtypes, often not further specified.

**Table 1:** Overview of studies regarding the presence of PD-1, PD-L1, and CD8 in AS

Study reference	*N*	AS subtype	Protein expression				Threshold for positivity			Antibodies			Correlation with prognosis
			PD-1 (%)	PD-L1 (%)	PD-1 andPD-L1 (%)	CD8 (%)	PD-1	PD-L1	CD8	PD-1	PD-L1	CD8	
*Kawamura *et al*. 2019 *[10]	29	Cutaneous	12 (41%)	22 (76%)	10 (34%)			> 5%^2^		Mouse anti-human ab (CST)	Rabbit anti-human ab (CST)		PD-L1 expression associated with poor survival
*Gambichler *et al*. 2020 *[11]	12	Cutaneous	4 (33%)	5 (42%)						Ab92484 (Abcam)	Ab205921 (Abcam)		
*Fujii *et al*. 2014 *[12]	55	Cutaneous (mainly UV)				20/40 (50%)			≥ Median/HPF			C8/144B (Dako)	High number of CD8 in primary tumor correlated with improved survival
*Honda *et al*. 2017 *[13]	106	Cutaneous (mainly UV)	19 (18%)	32 (30%)	9 (9%)		> 50/3HPF	> 5%^2^		NAT105 (Abcam)	SP142 (Spring Biosience)		PD-1 + cells (especially in combination with PD-L1 expression) correlated with improved survival
*Shimizu *et al*. 2017 *[14]	52	Cutaneous (mainly UV)		21 (40%)		24 (46%)		≥ 5%^2^	> Median		Rabbit monoclonal antibody (Abcam)	Abcam	PD-L1 and CD8 are associated with a worse outcome
*Bagaria *et al*. 2018 *[15]	26	Cutaneous (19), other (8)	1 (4%)^3^	5 (19%)^3^				≥ 5%^2^		NAT105 (Ventana)	SP-142 and SP-263 (Ventana)		No
*Botti *et al*. 2017 *[16]	24	Primary AS (7 breast, 5 soft tissue, 4 bone, 4 skin, 4 visceral)		16 (66%)				≥ 5%^2^			SP-142 (Spring Biosience)		No
*Googe *et al*. 2020 *[17]	10	Skin (9), soft tissue (1)	10 (100%)	10 (100%)	10 (100%)	10 (100%)		Low: ≥ 1% High: ≥ 50%		NAT105 (Cell Marque)	ZR 3 (Cell Marque)	4B11 (Leica Biosystems)	
*D’Angelo *et al*. 2015 *[18]	3	n.s		0 (0%)		1 (33%)		≥ 1%^2^	≥ 5%		Dako	C8/144B (Dako)	
*Kim *et al*. 2013 *[19]	5	n.s	4 (80%)	4 (80%)	4 (80%)		≥ 1	Total score ≥ 8^1^		NAT (Abcam)	H-130 (SCB)		
*Boxberg *et al*. 2018 *[20]	23	n.s		8 (35%) 4 (17%) 3 (13%) 2 (9%)				≥ 1%^2^ ≥ 5% ≥ 10% ≥ 50%			SP263 (Ventana)		
*Kosemehmetoglu *et al*. 2017 *[21]	7	n.s		1 (14%)				> 5%^2^			E1L3N (CST)		
*Que *et al*. 2017 *[22]	5	n.s		1 (20%)				> 1%^2^			E1L3N (CST)		
*Blessin *et al*. 2020 *[23]	25	n.s				Median count 95 cells/mm^2^						TC8 (ONCOdianova)	
*Orth *et al*. 2020 *[24]	6	n.s	2/5 (40%)	3 (50%)	1 (17%)		≥ 4/HPF	> 1%^2^		315 M (Cell Marque)	E1L3N (CST)		
*Vargas *et al*. 2020 *[25]	17	n.s		5 (29%)				≥ 1%^2^			SP263 (Ventana)		
*Lee *et al*. 2021 *[26]	70	n.s		13 (19%)				Combined positive score ≥ 1		22C3 (Agilent Technologies)			PD-L1 expression correlated with poor survival in metastatic AS patients
***Summary of results***^***4***^	**N**	**AS subtype**	**PD-1 expression (%)**	**PD-L1 expression (%)**	**PD-1 and PD-L1 expression (%)**	**CD8 expression (%)**	**PD-1 threshold**	**PD-L1 threshold**	**CD8 threshold**				
	286	Cutaneous	44/156 (28%)	92/212 (43%)	28/144 (19%)	53/101 (52%)	> 50/3HPF	≥ 1% to ≥ 5%	≥ Median				
	7	Breast		4/7 (57%)				≥ 5%					
	6	Soft tissue	1/1 (100%)	5/6 (83%)	1/1 (100%)	1/1 (100%)		≥ 1% to ≥ 5%					
	4	Bone		4/4 (100%)				≥ 5%					
	4	Visceral		1/4 (25%)				≥ 5%					
	169	n.s	6/10 (60%)	35/136 (26%)	5/11 (45%)	1/3 (33%)	≥ 1 to ≥ 4/HPF	≥ 1% to ≥ 8	≥ 5%				

Abbreviations: *AS*, angiosarcoma; *CST*, Cell Signaling Technology, *HPF*, high power field; *n.s.*, not specified; *SCB*, Santa Cruz Biotechnology; *UV*, UV-associated AS;

^1^For PD-L1, the staining intensity score was classified as 0 (no staining), 1 (weak staining), and 2 (intermediate staining), and 3 (strong staining). The area of staining was scored as 0 (0–10% of the cells stained), 1 (11–33% of the cells stained), 2 (34–66% of the cells stained), and 3 (67–100% of the cells stained). The total score was determined as the sum of the intensity score and the staining proportion score of two different TMAs. The total score ≥ 8 was determined as positive. ^2^Membranous expression of tumor cells. ^3^Expression not displayed per subtype. ^4^ Results are summarized per subtype

Clinical data with regard to the application of ICI in AS is limited to small case series. In one of these case series, three UV-associated AS were treated with anti-PD-1 (one in combination with CTLA-4 inhibition) showing partial response. One RT-associated AS also showed partial response (with anti-PD1) and one primary breast AS showed progressive disease on axitinib (VEGFR inhibitor) combined with PD-1 inhibition [27]. Four patients suffering from UV-associated AS with significant PD-L1 expression were successfully treated with anti-PD-1 [28–31]. In the recent AS patient-partnered-genomic study of Painter et al., a total of six patients were treated with anti-PD-1 [32]. Of these patients, two out of three UV-associated AS with high mutational burden (> 150 mutations/Mb) showed a complete response, whereas no clinical benefit was found in the other three patients (all non-UV AS with low mutational burden) [32]. Pecora et al. published a case of primary AS of the temple and one RT-associated AS with complete clinical remission on combined anti-CTLA4 and anti-PD-1 therapy (Pecora et al. CTOS2019).

Taken together, the previously mentioned studies suggest a potential therapeutic role for ICI in especially UV-associated AS. However, it yet remains unclear whether immunotherapy could be of interest for all AS patients or only for certain pathogenetic subgroups. Our recent DNA methylation profiling study confirmed the existence of these subgroups of AS on an epigenetic level, which did not fully match the clinical subtypes [33].

In the current retrospective study, we aimed to characterize PD-1, PD-L1 expression, and the presence of CD8 + T cells in a large cohort of AS tumor samples and their prognostic relevance to further explore the heterogeneity and the need to differentiate between the different AS subgroups.

## Materials and methods

### Tumor sample collection

We collected formalin-fixed paraffin-embedded (FFPE) tumor tissue of AS patients by a nationwide search through PALGA (Dutch nationwide network and registry of histo- and cytopathology) diagnosed between 1989 and 2015 in the Netherlands [34]. All cases were reviewed by an expert pathologist (UF), and confirmed AS cases were divided into pathogenetic subgroups based on available clinical data and pathology reports [35].

Tumor samples were collected on tissue microarrays (TMAs) and divided into the different subgroups, including 44 UV-associated, 14 cutaneous not UV-associated, 55 RT-associated, 14 Stewart Treves (lymphoedema-associated cases), 27 visceral, and 11 soft tissue cases. Only primary localized tumor samples were selected for this study. Of these samples, 33 have been previously subject to DNA methylation profiling [33].

### Clinical data

Clinical data were received from the nationwide Netherlands Cancer Registry and were linked to data from the Dutch pathology registry (PALGA). Ethical approval for the study was obtained from the local certified Medical Ethics Committee of the Radboudumc, Nijmegen, The Netherlands (file number 2016–2686).

### Immunohistochemistry

Immunohistochemical analysis was performed to investigate PD-1 and PD-L1 expression and the presence of CD8 + lymphocytes in the tumor. Tonsil (PD-L1 + , PD-1 + , and CD8 +) and appendix (CD8 + and PD1 +) served as positive controls. Immunohistochemistry was performed on 4-µm-thick FFPE sections of AS TMAs with one or two cores per sample from representative tumor areas (core size 2 mm) to allow simultaneous examination of patient specimens under identical conditions. Staining was performed in the Lab Vision Autostainer 360 (Thermo Fisher Scientific) by using the EnVision FLEX, pH high Link kit (Dako), and monoclonal rabbit anti-PD-L1 (1:800, clone E1L3N, Cell Signaling Technology), monoclonal mouse anti-PD-1 (1:20, clone MRQ-22, Cell Marque) or monoclonal mouse anti-CD8 (1:80, clone C8/144B, Dako).

PD-L1 expression on the tumor cells was scored as 0% ( −), 1–10% (+ / −), 10–50% ( +) or ≥ 50% positive tumor cells (+ +). All CD8 and PD-1 positive T cells were counted and subdivided in three categories: < 10 ( −), 10–50 ( +), or ≥ 50 positive cells (+ +) per tumor core [36].

Digital images were generated with VisionTekTM (Sakura, version 2.6) and analyzed at × 20 magnification.

### Statistical analysis

Statistical analyses were performed using IBM SPSS Statistics 25. *p*-values < 0.05 were considered significant and *p*-values < 0.1 were considered a trend. Relations between categorical parameters were assessed by chi-square or Fisher’s exact testing as appropriate, and associations with overall survival (OS) were assessed by the Kaplan–Meier method with the logrank test. Tumors positive for one marker or a combination of markers were compared to tumors negative for that particular marker or combination. A distinction between the different levels of expression was made in the analysis.

## Results

### Immune profiles in AS subtypes

The expression of PD-1, PD-L1, and CD8 was assessed in 165 AS samples divided over 6 different subgroups. Patient characteristics are shown in Table 2. Staining results are presented in Table 3 with an example of each staining shown in Supplementary Fig. 1.

**Table 2:** Patient characteristics

	*N* (%)
**AS samples**	**165**
UV associated	44 (27)
Cutaneous not UV associated	14 (8)
RT associated	55 (33)
Stewart Treves	14 (8)
Visceral	27 (16)
Soft tissue	11 (7)
**Extent of disease**	
Localized	80 (48)
Invasion adjacent structures	9 (5)
Lymph node involvement	4 (2)
Distant metastases	14 (8)
Unknown	58 (35)
**Tumor depth**	
Superficial	44 (27)
Deep	5 (3)
Unknown	116 (70)
**Distant metastases**	
No	111 (67)
Yes	20 (12)
Unknown	34 (21)
**Age**	
< 40	6 (4)
≥ 40 < 70	55 (33)
≥ 70	104 (63)
**Gender**	
Male	54 (33)
Female	111 (67)
**Follow-up status** (median follow-up 14.8 months)	
Alive	20 (12)
Deceased	145 (88)

**Table 3:** PD-L1, PD-1 expression, and the presence of CD8 + T cells in angiosarcoma

AS subgroup	*N*	PD-L1 ≥ 1%	PD-L1 ≥ 10%	PD-L1 ≥ 50%	PD-1 ≥ 10	PD1 ≥ 50	CD8 ≥ 10	CD8 ≥ 50	PD-1 and PD-L1 ≥ 10 (%)	PD-1 and PD-L1 ≥ 50 (%)
UV associated	44	89%	66%	37%	66%	39%	98%	79%	50%	18%
Cutaneous not UV	14	86%	50%	7%	64%	7%	93%	64%	21%	0%
RT associated	55	79%	52%	8%	68%	32%	98%	81%	39%	6%
Stewart Treves	14	75%	50%	8%	43%	7%	100%	86%	36%	0%
Visceral	27	79%	54%	46%	52%	20%	92%	69%	38%	17%
Soft tissue	11	100%	78%	56%	72%	36%	91%	82%	60%	40%

High PD-1 and PD-L1 expressions were predominantly seen in clinically defined soft tissue (40%), UV-associated (18%), and visceral (17%) AS subgroups. Besides, RT-associated AS showed predominantly high PD-1 expression (32%). Infiltration of high numbers of CD8 + T cells was present in the majority of AS samples across all different subgroups (64–86%) (Table 3).

**Fig. 1:**
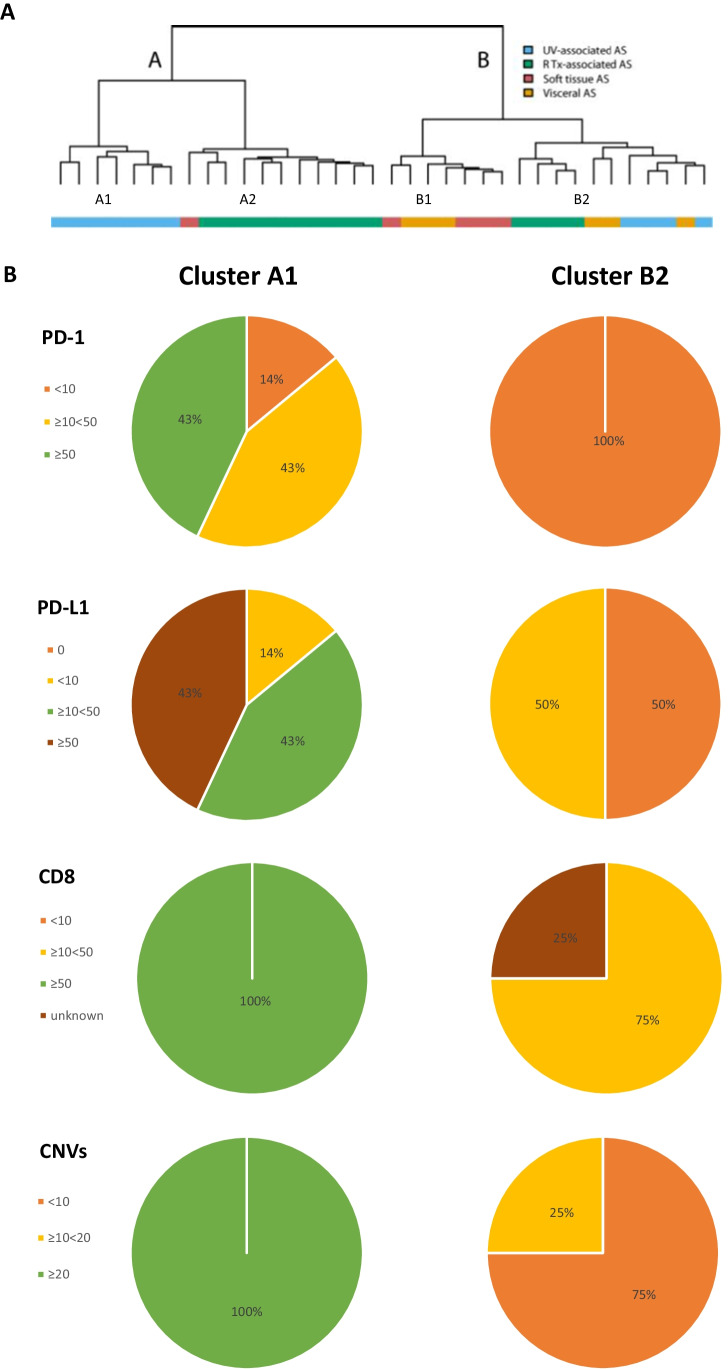
Overview of the division of AS subgroups over the different methylation clusters (**A**). Expression of PD-1, PD-L1, CD8, and the presence of copy number variations (CNVs) in UV-associated AS in cluster A1 versus B2 (**B**)

The two main clusters (A and B) defined by our previous genome-wide array-based DNA methylation profiling study were each subdivided into 2 separate clusters (A1, A2 and B1, B2) (Fig. 1A). Cluster A1 consisted exclusively of UV-associated cases, whereas A2 primarily consisted of RT-associated cases. Cluster B1 had both visceral and soft tissue cases, and cluster B2 was mixed, including cases of UV-associated AS.

In the current study, a significantly higher PD-L1 expression (≥ 10%) and PD-1 expression (≥ 10) was found in the UV-associated cases in cluster A1 versus those in cluster B2 (for both stainings 6/7 (86%) in A1 versus 0/4 (0%) in B2, *p* = 0.015) (Fig. 1B).

A high amount of CD8-positive T cells (≥ 50) was observed in all UV-associated cases in cluster A1 versus none of the UV-associated cases in cluster B2 (7/7 (100%) in A1 versus 0/3 (0%) in B2 (1 case was not evaluable), *p* = 0.008) (Fig. 1B). A difference in the number of copy number variations (CNVs) was seen between the UV-associated AS cases in cluster A1 versus B2, as already described in our methylation profiling study (mean number of CNVs 34.4 (range 23–39) in cluster A1 versus 7.3 (range 3–17) in cluster B2, *p* < 0.001) (Fig. 1B).

For the other AS subgroups, no significant differences in expression of PD-L1, PD-1, or CD8 were observed between clusters (Supplementary Fig. 2).

### Prognostic relevance of the immune profile

**Fig. 2:**
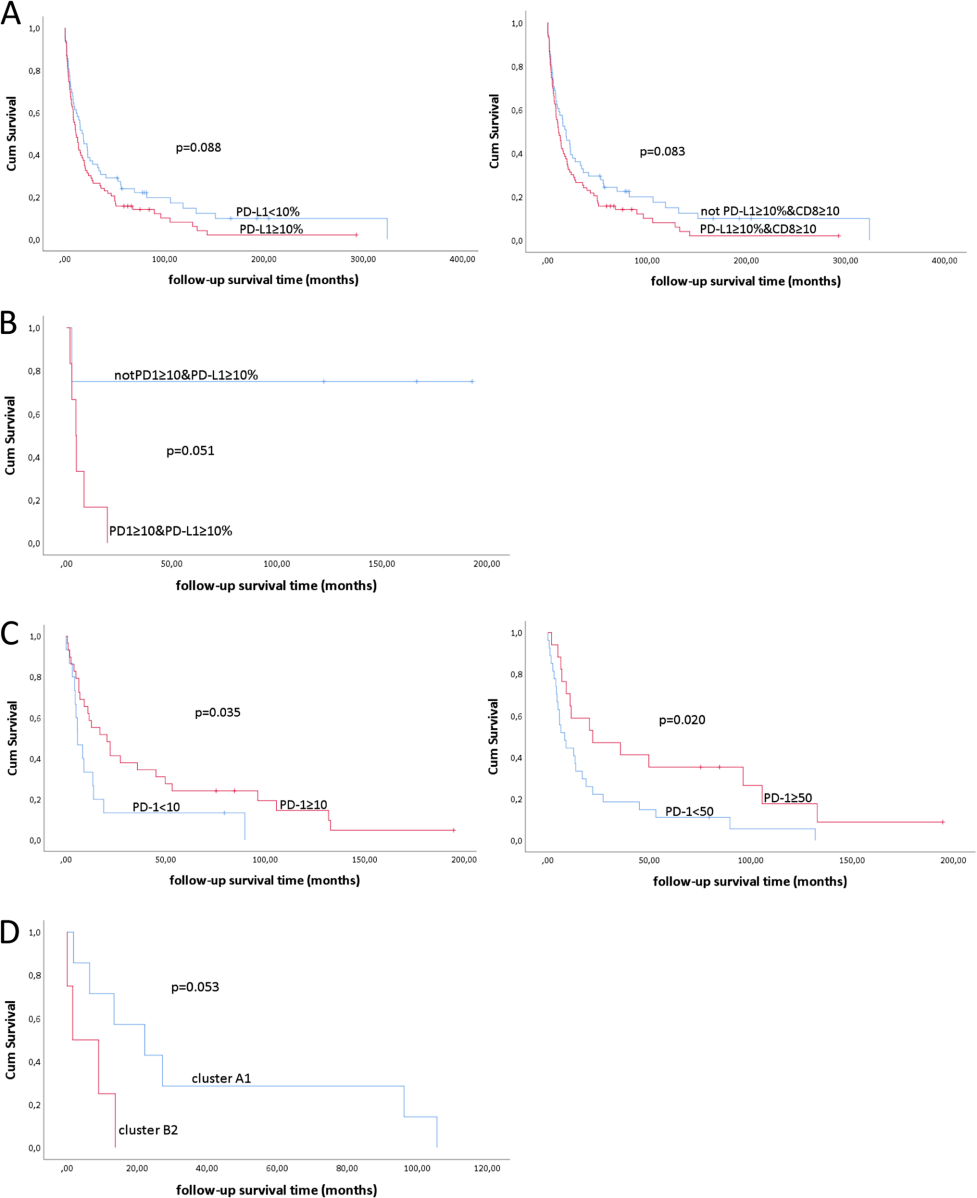
Kaplan–Meier curves showing the significant differences and trends in overall survival in **A** AS total group, **B** soft tissue AS, and **C** UV-associated AS according to PD-1, PD-L1 expression, or the presence of CD8 + T cells in the tumor and a Kaplan–Meier curve showing the difference in overall survival between UV-associated AS in cluster A1 versus B2 (**D**)

In Supplementary Table 1, we present the univariate analysis of associations of the expression of the different markers with overall survival. In the total group PD-1, PD-L1, CD8, or combined expression did not significantly correlate with survival, although we did observe a trend toward a worse overall survival for patients with ≥ 10% PD-L1 expression in their tumor versus < 10% PD-L1 (median 11.0 ± 2.4 versus 17.1 ± 4.1 months, respectively, *p* = 0.088) and for patients with both ≥ 10% PD-L1 expression and ≥ 10 CD8-positive T cells in their tumor versus those with no expression of both markers (median 11.0 ± 2.4 months versus 18.4 ± 4.1 months, respectively, *p* = 0.083) (Fig. 2A).

In the cutaneous not UV-associated, RT-associated, Stewart Treves, and visceral AS groups, no significant correlations with survival were observed. We excluded correlations when groups of only one patient were involved.

In patients with soft tissue AS, the presence of both PD-1 (≥ 10) and PD-L1 (≥ 10%) showed a trend toward poor survival (estimate mean survival time 6.8 ± 2.7 months (both positive, *n* = 6) versus 145.5 ± 41.3 months (not both positive, *n* = 4), *p* = 0.051) (Fig. 2B).

In UV-associated AS, the presence of PD-1 positive cells in the tumor (≥ 10) correlated with better overall survival (median 20.6 ± 8.5 months for PD1 ≥ 10 versus 5.8 ± 2.1 months for PD1 < 10, p = 0.035). The same applies to the presence of high numbers of PD-1 positive cells in the tumor (≥ 50) (median 22.1 ± 16.7 months for PD1 ≥ 50 versus 8.3 ± 2.8 months for PD1 < 50, *p* = 0.020) (Fig. 2C). All PD-1 positive tumors in this subgroup were also CD8 positive.

UV-associated AS patients in cluster A1 (instable, more immunogenic (“hot”) cluster, *n* = 7) showed a trend toward better overall survival compared to those in cluster B2 (stable, “cold” cluster, *n* = 4) (median 22.2 ± 11.4 versus 1.6 ± 4.5 months, *p* = 0.053) (Fig. 2D).

### Correlations with patient characteristics

Correlations with gender, age, tumor depth, and presence of distant metastases are presented in Table 4. In the total group, PD-L1 expression (≥ 10%) already showed a trend toward a correlation with the male gender (*p* = 0.075), whereas high PD-L1 expression (≥ 50%) correlated significantly with the male gender (17/47 (36%) male versus 19/98 (19%) female, *p* = 0.039). Also, combined positive PD-L1 and CD8 (≥ 10(%)) expression and high PD-L1 and CD8 (≥ 50(%)) expression showed a trend toward a correlation with the male gender (*p* = 0.070/0.075). High PD-L1 expression (≥ 50%) was also more common in deep tumors (3/4 (75%) deep versus 6/40 (15%) superficial tumors, *p* = 0.023).

**Table 4:** Correlations between (combinations of) biomarkers and clinical data

AS subgroup	Clinical marker	PD-L1			PD-1		CD8		PD-L1 and PD-1		PD-L1 and CD8		PD-1 and CD8		PD-L1 and PD-1 and CD8	
		**1**	**10**	**50**	**10**	**50**	**10**	**50**	**10**	**50**	**10**	**50**	**10**	**50**	**10**	**50**
All	Gender	-	0.075	**0.039**	-	-	-	-	-	-	0.070	0.075	-	-	-	-
	Age	-	-	-	-	-	-	-	-	-	-	-	-	-	-	-
	Tumor depth	-	-	**0.023**	-	-	-	-	-	-	-	-	-	-	-	-
	Distant metastases	-	-	-	-	-	-	-	-	-	-	-	-	-	-	-
UV associated	Gender	-	-	-	-	-	-	-	-	-	-	-	-	-	-	-
	Age	-	-	-	-	-	-	-	-	-	-	-	-	-	-	-
	Tumor depth	-	-	-	-	-	-	-	-	-	-	-	-	-	-	-
	Distant metastases	-	-	-	-	-	-	-	-	-	-	-	-	-	-	-
Cutaneous not UV	Gender	-	-	-	-	-	-	-	-	-	-	-	-	-	-	-
	Age	-	-	-	-	-	-	-	-	-	-	-	-	-	-	-
	Tumor depth	-	-	-	-	-	-	-	-	-	-	-	-	-	-	-
	Distant metastases	-	-	-	-	-	-	-	-	-	-	-	-	-	-	-
RT associated	Gender	-	-	-	-	-	-	-	-	-	-	-	-	-	-	-
	Age	-	-	**0.031**	-	-	-	-	-	0.077	-	**0.031**	-	-	-	0.077
	Tumor depth	-	-	-	-	-	-	-	-	-	-	-	-	-	-	-
	Distant metastases	0.066	-	-	-	**0.034**	-	-	-	-	-	-	-	**0.034**	-	-
Stewart Treves	Gender	-	-	-	-	-	-	-	-	-	-	-	-	-	-	-
	Age	-	-	-	-	-	-	-	-	-	-	-	-	-	-	-
	Tumor depth	-	-	-	-	-	-	-	-	-	-	-	-	-	-	-
	Distant metastases	-	-	-	-	-	-	-	-	-	-	-	-	-	-	-
Visceral	Gender	-	-	-	-	-	-	-	-	-	-	-	-	-	-	-
	Age	-	-	-	-	-	-	-	-	-	-	-	-	-	-	-
	Tumor depth	-	-	-	-	-	-	-	-	-	-	-	-	-	-	-
	Distant metastases	-	-	**0.036**	-	-	-	-	-	-	-	-	-	-	-	-
Soft tissue	Gender	-	-	-	-	-	-	-	-	-	-	-	-	-	-	-
	Age	-	-	-	-	-	-	-	-	-	-	-	-	-	-	-
	Tumor depth	-	-	-	-	-	-	-	-	-	-	-	-	-	-	-
	Distant metastases	-	-	-	-	-	-	-	-	-	-	-	-	-	-	-

*p*-value < 0.05 is considered significant (*p*-value shown in bold), *p*-value < 0.1 is considered a trend (*p*-value shown);—means no significant correlation. Tumor depth compares deep and superficial tumors, age compares patients < 70 years of age with patients ≥ 70 years of age

In RT-associated AS, PD-L1 expression (≥ 1%) showed a trend toward a correlation with the absence of metastases (*p* = 0.066), whereas no significant correlation with the presence of distant metastases was observed at the thresholds of 10 or 50%. High PD-L1 expression (≥ 50%) did show a significant correlation with the age below 70 years (4/21 (19%) < 70 years of age versus 0/27 (0%) ≥ 70 years of age, *p* = 0.031). All cases with high PD-L1 expression also showed a high CD8 expression. The combination of both high PD-L1 and PD-1 expressions, as well as the combination of high PD-L1, PD-1, and CD8 expressions showed a trend toward a correlation with the age group below 70 years (*p* = 0.077). High PD-1 expression (≥ 50) correlated with the presence of distant metastases (PD-1 ≥ 50 in 3/3 (100%) patients with distant metastases versus 11/27 (41%) without distant metastases, *p* = 0.034). Cases with high PD-1 expression also showed high CD8 expression.

In visceral AS, high PD-L1 expression (≥ 50%) correlated with the absence of distant metastases (PD-L1 ≥ 50% in 0/5 (0%) patients with distant metastases versus 8/13 (62%) without distant metastases, *p* = 0.036).

## Discussion

This is the first study mapping the immunological landscape in different AS subgroups as well as in genome-wide methylation profiling clusters. We detected relevant differences between the various subgroups.

We showed a high expression of both PD-1 and PD-L1 predominantly in UV-associated, visceral, and soft tissue AS subgroups and high PD-1 expression in RT-associated AS, whereas infiltration of CD8 + T cells was present in the majority of AS samples. In soft tissue AS the presence of both PD-1 and PD-L1 expression showed a trend toward poor survival, whereas in UV-associated AS, PD-1 expression was correlated with better survival.

These results reflect the heterogeneity in immunological response associated with prognosis for the diverse AS subgroups and underline the need to differentiate between them. So far, most studies have analyzed PD-1, PD-L1, and CD8 expression only in cutaneous, mainly UV-associated AS (displayed in Table 1). Based on the observed high expression of PD-1, PD-L1, and CD8 in visceral and soft tissue AS in the current study, ICI might also be successful in these subgroups. Individual immune profiling before the start of ICI could be considered to select more vulnerable tumors.

In UV-associated AS, some studies reveal a correlation between PD-L1 positivity and worse prognosis and/or tumor cell proliferation [10, 14]. Honda et al. found an association between high infiltration of PD-1 positive cells and favorable survival [13]. This is in accordance with our results. One explanation could be that PD-1 expression might reflect antitumor immune response instead of tumor evasion. This indicates a response of the tumor cell to high immune pressure by CD8 + T cells and could lead to a better prognosis as long as the balance is on the side of antitumor immunity [37, 38].

Our finding that UV-associated AS may be classified in “cold” (low number of CD8 + T cells (< 50) and low or no PD-1 / PD-L1 expression (< 10(%)) and “hot” AS (high number of CD8 + T cells (≥ 50) and high PD-1/PD-L1 expression (≥ 10(%))) according to their methylation profile reflects with the data of Chan et al. who also showed immunologically “cold” and “hot” clusters within the UV-associated and other cutaneous AS of the head and neck (*n* = 13) based on NanoString profiling [39]. The “hot” tumors are expected to benefit from ICI; however, to draw definitive conclusions, a thorough investigation is necessary.

Recent studies have however shown that expression levels of PD-1, PD-L1, and CD8 on their own might not be sufficient to predict response to ICI [40, 41]. Other factors that may be important are tumor mutational burden (TMB), inflammation, and the further composition of the tumor immune microenvironment [42, 43]. It will be of (therapeutic) interest whether these markers also play a role in angiosarcomas.

In the study of Chan et al., cases with high TMB (*n* = 3) were all present in the immune “hot” cluster [39]. According to our copy number variation data in our previous methylation study, the immune “hot” UV-associated cluster appears to be the population harboring chromosomal instability [33]. Similar to tumor mutational burden, chromosomal instability might also reflect the neoantigen load of the tumor that mediates T cell responses against the tumor [44].

A very recent study suggests the use of tumor DNA methylation profiles to predict the response to anti-PD1 inhibitors in sarcomas [40]. They included only 2 AS patients (1 breast, 1 chest wall) who did not respond to the anti-PD1 treatment. Although we do not know if the patients in our study respond to ICI, we do see a difference in methylation profiles within the UV-associated AS which corresponds to different immune profiles.

In order to generate a more robust way to predict the response to ICI, it is necessary to analyze and combine multiple biomarkers and validate those in a large clinical trial.

It is remarkable that all previous studies chose a cutoff value of ≥ 1% or ≥ 5% for PD-L1 positive staining. Unlike the other studies, we performed statistical analyses on PD-L1 using different cutoff values (≥ 1%, ≥ 10%, and ≥ 50%). We found no statistically relevant correlations using a cutoff value of ≥ 1%. This may suggest that in angiosarcomas, PD-L1 as a prognostic marker is more valuable using a cutoff value of ≥ 10% or ≥ 50% compared to ≥ 1%. The optimal cutoff value for PD-L1 is still unknown and seems to be tumor-specific and even antibody-specific [45–47]. Therefore, the optimal PD-L1 cutoff value still needs to be evaluated. Furthermore, in epithelial cancer, different scores are established for PD-L1, including tumor proportion score (TPS), combined positivity score (CPS), and immune cell (IC) infiltrate [48]. In this paper, we only determined TPS. The right scoring system for AS is not yet established. Although it would make sense to add the PD-L1 positive immune cells instead of focusing only on the tumor cells, we also feel that manual counting on a stained slide without other markers to distinguish the type of immune cells is not enough. It would make sense to determine these scores in a future study by using multiplex immunostaining and quantifying different types of immune cells and their PD-L1 expression in a large collection of angiosarcomas.

In conclusion, with this retrospective immunohistochemical study, we present evidence of subgroup-associated immune profiles of AS corresponding to pathogenesis, prognosis, and epigenetic mechanisms. We showed that expression of the immunological markers PD-1 and PD-L1 was clearly present in several AS subgroups besides cutaneous (UV-associated) AS, with varying prognostic correlations. We confirmed the existence of two different clusters within the UV-associated subgroup, revealing one immunologically “hot” (chromosomally unstable) and one “cold” (chromosomally stable) cluster. Given the scarce treatment options in AS, our results provide a rationale for the future investigation/application of immune checkpoint inhibition in AS.

## Supplementary Information

Below is the link to the electronic supplementary material.

Supplementary file1 (DOCX 19 KB)

Supplementary Figure 1. Examples of PD-L1 expression on AS tumor cells (A), PD-1 expression on T cells (B) and CD8 positive T cells in AS (C). Images were taken at 20x magnification. Supplementary Figure 2. Differences in expression of PD-L1, PD-1 and CD8 per subgroup between clusters. RT-associated cases were divided in cluster A2 (n=10) and B2 (n=4), soft tissue AS were divided in cluster A2 (n=1) and B1 (n=4), and visceral AS were divided in cluster B1 (n=3) and B2 (n=3). (PDF 293 KB)

## Data Availability

Data and material are available upon request.
