# Supplementary material for: Analysis of PD-1, PD-L1, and T-cell infiltration in angiosarcoma pathogenetic subgroups
Source: Immunol Res. 2022 Jan 19;70(2):256–68. doi: 10.1007/s12026-021-09259-4 (PMC8916989; doi:10.1007/s12026-021-09259-4)
Supplement: Supplementary file 1 — Supplementary file1 (DOCX 19 KB) [file 12026_2021_9259_MOESM1_ESM.docx]

**Supplemental Table 1 Univariate analyses of marker associations with overall survival in AS patients**

| AS subgroup | variables | Univariate analysis | | |
| --- | --- | --- | --- | --- |
|  |  | OS (months) | | P value |
| All | PD-L1 (<1% vs ≥1%) | 15.2 vs 12.6 | - | |
|  | PD-L1 (<10% vs ≥10%) | 17.1 vs 11.0 | 0.088 | |
|  | PD-L1 (<50% vs ≥50%) | 13.8 vs 12.7 | - | |
|  | PD-1 (<10 vs ≥10) | 10.8 vs 15.2 | - | |
|  | PD-1 (<50 vs ≥50) | 13.7 vs 15.8 | - | |
|  | CD8 (<10 vs ≥10) | 9.7 vs 13.7 | - | |
|  | CD8 (<50 vs ≥50) | 8.2 vs 15.8 | - | |
|  | PD-L1&PD-1 (<10 vs ≥10) | 14.8 vs 12.6 | - | |
|  | PD-L1&PD-1 (<50 vs ≥50) | 13.5 vs 11.6 | - | |
|  | PD-L1&CD8 (<10 vs ≥10) | 18.4 vs 11.0 | 0.083 | |
|  | PD-L1&CD8 (<50 vs ≥50) | 12.6 vs 13.4 | - | |
|  | PD-1&CD8 (<10 vs ≥10) | 10.8 vs 15.2 | - | |
|  | PD-1&CD8 (<50 vs ≥50) | 13.7 vs 15.8 | - | |
|  | PD-L1&PD1&CD8 (<10 vs ≥10) | 14.8 vs 12.6 | - | |
|  | PD-L1&PD1&CD8 (<50 vs ≥50) | 13.4 vs 11.6 | - | |
| UV-associated | PD-L1 (<1% vs ≥1%) | 4.3 vs 11.6 | - | |
|  | PD-L1 (<10% vs ≥10%) | 9.0 vs 12.7 | - | |
|  | PD-L1 (<50% vs ≥50%) | 6.9 vs 13.4 | - | |
|  | PD-1 (<10 vs ≥10) | 5.8 vs 20.6 | **0.035** | |
|  | PD-1 (<50 vs ≥50) | 8.3 vs 22.1 | **0.020** | |
|  | CD8 (<10 vs ≥10) | 19.0 vs 11.0 | - | |
|  | CD8 (<50 vs ≥50) | 5.8 vs 12.7 | - | |
|  | PD-L1&PD-1 (<10 vs ≥10) | 8.3 vs 17.0 | - | |
|  | PD-L1&PD-1 (<50 vs ≥50) | 9.0 vs 20.6 | - | |
|  | PD-L1&CD8 (<10 vs ≥10) | 5.8 vs 12.7 | - | |
|  | PD-L1&CD8 (<50 vs ≥50) | 6.9 vs 13.4 | - | |
|  | PD-1&CD8 (<10 vs ≥10) | 5.7 vs 20.6 | **0.035** | |
|  | PD-1&CD8 (<50 vs ≥50) | 6.4 vs 22.1 | **0.020** | |
|  | PD-L1&PD1&CD8 (<10 vs ≥10) | 5.8 vs 17.0 | - | |
|  | PD-L1&PD1&CD8 (<50 vs ≥50) | 8.3 vs 20.6 | - | |
| Cutaneous not UV | PD-L1 (<1% vs ≥1%) | 11.3 vs 3.6 | - | |
|  | PD-L1 (<10% vs ≥10%) | 11.3 vs 7.9 | - | |
|  | PD-L1 (<50% vs ≥50%) | 7.9 vs 49.9^3^ | - | |
|  | PD-1 (<10 vs ≥10) | 3.6 vs 11.3 | - | |
|  | PD-1 (<50 vs ≥50) | 8.1 vs 7.9^3^ | - | |
|  | CD8 (<10 vs ≥10) | 1.6 vs 8.1^3^ | 0.097 | |
|  | CD8 (<50 vs ≥50) | 8.1 vs 7.9 | - | |
|  | PD-L1&PD-1 (<10 vs ≥10) | 8.1 vs 7.9 | - | |
|  | PD-L1&PD-1 (<50 vs ≥50) | n.a.^2^ | n.a. | |
|  | PD-L1&CD8 (<10 vs ≥10) | 11.3 vs 7.9 | - | |
|  | PD-L1&CD8 (<50 vs ≥50) | n.a.^2^ | n.a. | |
|  | PD-1&CD8 (<10 vs ≥10) | 3.6 vs 11.3 | - | |
|  | PD-1&CD8 (<50 vs ≥50) | 8.1 vs 7.9^3^ | - | |
|  | PD-L1&PD1&CD8 (<10 vs ≥10) | 8.1 vs 7.9 | - | |
|  | PD-L1&PD1&CD8 (<50 vs ≥50) | n.a.^2^ | n.a. | |
| RT-associated | PD-L1 (<1% vs ≥1%) | 22.8 vs 22.5 | - | |
|  | PD-L1 (<10% vs ≥10%) | 25.1 vs 19.4 | - | |
|  | PD-L1 (<50% vs ≥50%) | 22.8 vs 15.8 | - | |
|  | PD-1 (<10 vs ≥10) | 36.1 vs 22.5 | - | |
|  | PD-1 (<50 vs ≥50) | 25.1 vs 18.1 | - | |
|  | CD8 (<10 vs ≥10) | 32.9 vs 22.8^3^ | - | |
|  | CD8 (<50 vs ≥50) | 22.8 vs 23.5 | - | |
|  | PD-L1&PD-1 (<10 vs ≥10) | 25.1 vs 19.4 | - | |
|  | PD-L1&PD-1 (<50 vs ≥50) | 22.8 vs 18.1 | - | |
|  | PD-L1&CD8 (<10 vs ≥10) | 25.1 vs 19.4 | - | |
|  | PD-L1&CD8 (<50 vs ≥50) | 22.8 vs 15.8 | - | |
|  | PD-1&CD8 (<10 vs ≥10) | 36.1 vs 22.5 | - | |
|  | PD-1&CD8 (<50 vs ≥50) | 25.1 vs 18.1 | - | |
|  | PD-L1&PD1&CD8 (<10 vs ≥10) | 25.1 vs 19.4 | - | |
|  | PD-L1&PD1&CD8 (<50 vs ≥50) | 22.8 vs 18.1 | - | |
| Stewart Treves | PD-L1 (<1% vs ≥1%) | 34.1 vs 10.8 | - | |
|  | PD-L1 (<10% vs ≥10%) | 18.4 vs 10.3 | - | |
|  | PD-L1 (<50% vs ≥50%) | 17.1 vs 68.3^3^ | - | |
|  | PD-1 (<10 vs ≥10) | 18.4 vs 9.8 | - | |
|  | PD-1 (<50 vs ≥50) | 18.4 vs 0.3^3^ | **0.000** | |
|  | CD8 (<10 vs ≥10) | n.a.^2^ | n.a. | |
|  | CD8 (<50 vs ≥50) | 2.5 vs 17.1 | - | |
|  | PD-L1&PD-1 (<10 vs ≥10) | 18.4 vs 10.3 | - | |
|  | PD-L1&PD-1 (<50 vs ≥50) | n.a.^2^ | n.a. | |
|  | PD-L1&CD8 (<10 vs ≥10) | 18.4 vs 10.3 | - | |
|  | PD-L1&CD8 (<50 vs ≥50) | 17.1 vs 68.3^3^ | - | |
|  | PD-1&CD8 (<10 vs ≥10) | 18.4 vs 9.8 | - | |
|  | PD-1&CD8 (<50 vs ≥50) | 18.4 vs 0.3^3^ | **0.000** | |
|  | PD-L1&PD1&CD8 (<10 vs ≥10) | 18.4 vs 10.3 | - | |
|  | PD-L1&PD1&CD8 (<50 vs ≥50) | n.a.^2^ | n.a. | |
| Visceral | PD-L1 (<1% vs ≥1%) | 2.2 vs 3.4 | - | |
|  | PD-L1 (<10% vs ≥10%) | 5.1 vs 2.1 | - | |
|  | PD-L1 (<50% vs ≥50%) | 2.2 vs 3.4 | - | |
|  | PD-1 (<10 vs ≥10) | 5.1 vs 2.1 | - | |
|  | PD-1 (<50 vs ≥50) | 5.1 vs 2.1 | - | |
|  | CD8 (<10 vs ≥10) | 0.0 vs 3.4 | - | |
|  | CD8 (<50 vs ≥50) | 1.6 vs 5.1 | - | |
|  | PD-L1&PD-1 (<10 vs ≥10) | 5.1 vs 2.1 | - | |
|  | PD-L1&PD-1 (<50 vs ≥50) | 2.2 vs 2.1 | - | |
|  | PD-L1&CD8 (<10 vs ≥10) | 5.1 vs 2.1 | - | |
|  | PD-L1&CD8 (<50 vs ≥50) | 1.6 vs 3.4 | - | |
|  | PD-1&CD8 (<10 vs ≥10) | 5.1 vs 2.1 | - | |
|  | PD-1&CD8 (<50 vs ≥50) | 5.1 vs 2.1 | - | |
|  | PD-L1&PD1&CD8 (<10 vs ≥10) | 3.3 vs 0.8 | - | |
|  | PD-L1&PD1&CD8 (<50 vs ≥50) | 2.2 vs 2.1 | - | |
| Soft tissue | PD-L1 (<1% vs ≥1%) | n.a.^2^ | n.a. | |
|  | PD-L1 (<10% vs ≥10%) | n.a.^1,3^ | **0.030** | |
|  | PD-L1 (<50% vs ≥50%) | 4.6 vs 4.4 | - | |
|  | PD-1 (<10 vs ≥10) | n.a | - | |
|  | PD-1  (<50 vs ≥50) | 13.1 vs 4.4 | - | |
|  | CD8 (<10 vs ≥10) | n.a.^1,2^ | - | |
|  | CD8 (<50 vs ≥50) | 2.4 vs 8.3 | - | |
|  | PD-L1&PD-1 (<10 vs ≥10) | n.a. | 0.051 | |
|  | PD-L1&PD-1 (<50 vs ≥50) | 4.6 vs 4.4 | - | |
|  | PD-L1&CD8 (<10 vs ≥10) | n.a.^1,3^ | **0.030** | |
|  | PD-L1&CD8 (<50 vs ≥50) | 4.6 vs 4.4 | - | |
|  | PD-1&CD8 (<10 vs ≥10) | n.a. | - | |
|  | PD-1&CD8 (<50 vs ≥50) | 13.1 vs 4.4 | - | |
|  | PD-L1&PD1&CD8 (<10 vs ≥10) | n.a. | - | |
|  | PD-L1&PD1&CD8 (<50 vs ≥50) | 4.6 vs 4.4 | - | |
|  |  |  |  | |

p-value <0.05 is considered significant (p-value shown in bold), p-value <0.1 is considered a trend (p-value shown), - means no significant correlation. ^1^all cases censored, ^2^groups included with n=0, ^3^groups included with n=1, n.a. is not analyzed.
