# Supplementary figures and images for: Analysis of PD-1, PD-L1, and T-cell infiltration in angiosarcoma pathogenetic subgroups
Source: Immunol Res. 2022 Jan 19;70(2):256–68. doi: 10.1007/s12026-021-09259-4 (PMC8916989; doi:10.1007/s12026-021-09259-4)

## Supplemental Figure 1

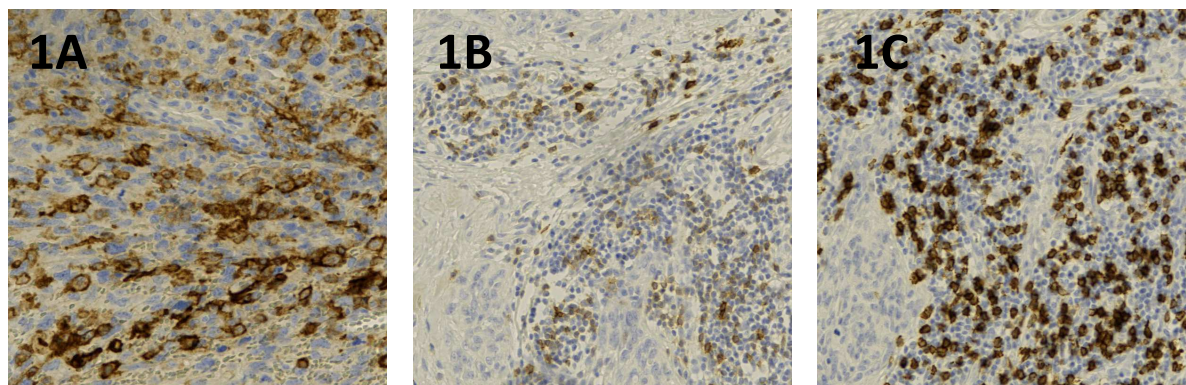

### RT associated AS

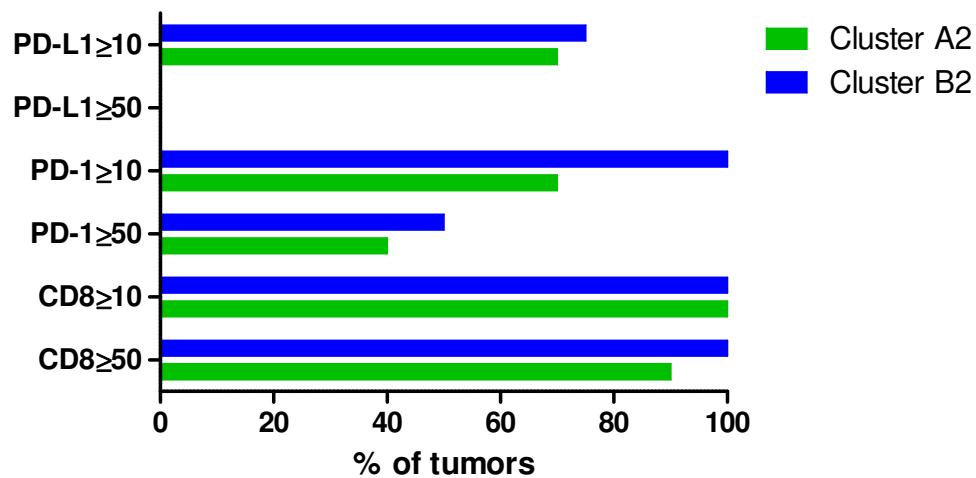

### Soft tissue AS

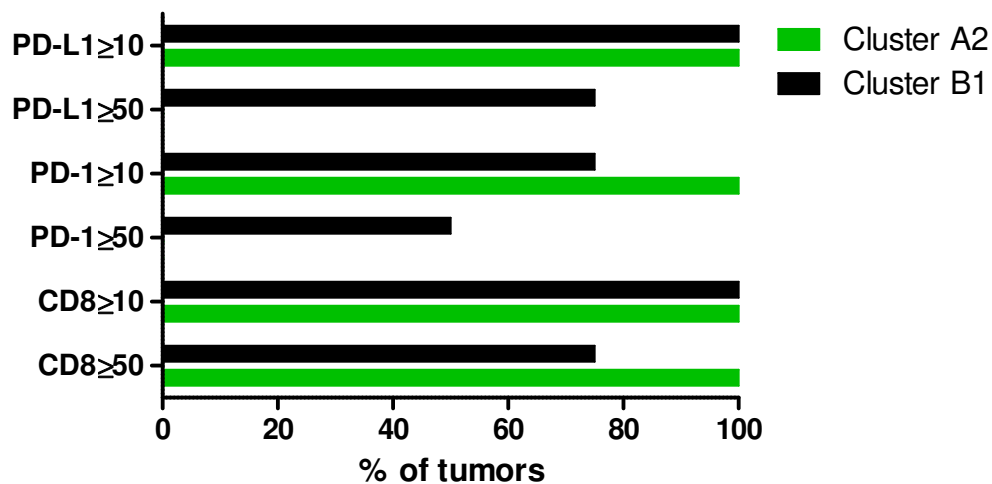

### Visceral AS

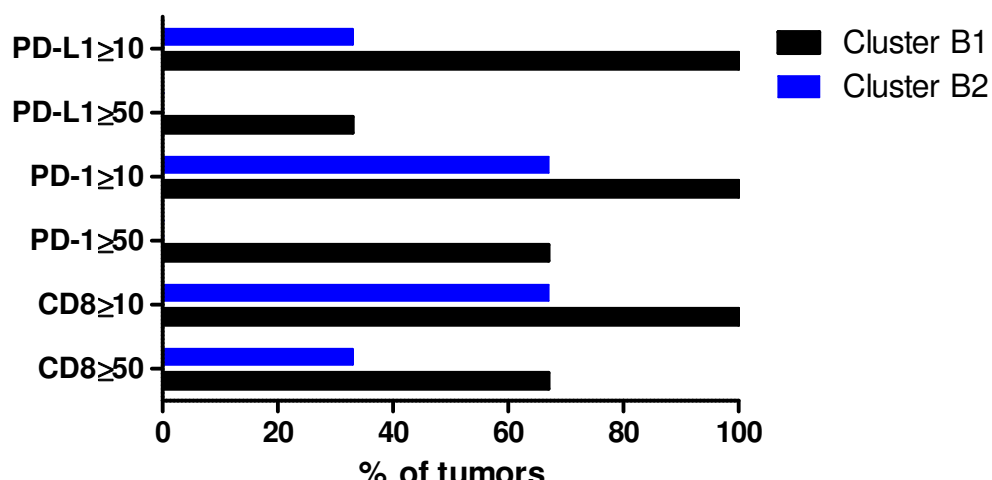

Supplement: Supplementary file 2 — Supplementary Figure 1. Examples of PD-L1 expression on AS tumor cells (A), PD-1 expression on T cells (B) and CD8 positive T cells in AS (C). Images were taken at 20x magnification. Supplementary Figure 2. Differences in expression of PD-L1, PD-1 and CD8 per subgroup between clusters. RT-associated cases were divided in cluster A2 (n=10) and B2 (n=4), soft tissue AS were divided in cluster A2 (n=1) and B1 (n=4), and visceral AS were divided in cluster B1 (n=3) and B2 (n=3). (PDF 293 KB) [file 12026_2021_9259_MOESM2_ESM.pdf]
